# Sound and Complete Concolic Testing for Higher-order Functions

**DOI:** 10.1007/978-3-030-72019-3_23

**Published:** 2021-03-23

**Authors:** Shu-Hung You, Robert Bruce Findler, Christos Dimoulas

**Affiliations:** Imperial College, London, UK; Northwestern University, Evanston, IL USA

## Abstract

Higher-order functions have become a staple of modern programming languages. However, such values stymie concolic testers, as the SMT solvers at their hearts are inherently first-order.

This paper lays a formal foundations for concolic testing higher-order functional programs. Three ideas enable our results: (i) our tester considers only program inputs in a canonical form; (ii) it collects novel constraints from the evaluation of the canonical inputs to search the space of inputs with partial help from an SMT solver and (iii) it collects constraints from canonical inputs even when they are arguments to concretized calls. We prove that (i) concolic evaluation is sound with respect to concrete evaluation; (ii) modulo concretization and SMT solver incompleteness, the search for a counter-example succeeds if a user program has a bug and (iii) this search amounts to directed evolution of inputs targeting hard-to-reach corners of the program.

### 1 Introduction

Concolic testing [8, 20] allows symbolic evaluation to leverage concrete inputs as it attempts to uncover bugs. The role of concrete inputs is twofold. First, they help symbolic evaluation focus on one control-flow path at a time, thus allowing the exploration of the behavior of a user program in an incremental and directed fashion. Second, they enable concretization, permitting symbolic evaluation to seamlessly switch to concrete evaluation and back, thus facilitating interoperability with external libraries. Testament to the success of concolic testing is adaptations to a gamut of linguistic, platform and application settings [3, 6, 7, 12, 14, 15, 17, 21, 22, 23, 25, 29, 30, 35, 37, 38, 39, 41, 43].

However, concolic testers' generation of inputs hinges on the power of SMT solvers. That is, at the end of a run of a user program, the concolic tester constructs a formula whose solution determines the next input. Alas, SMT solvers largely deal with first-order formulas that cannot capture higher-order properties of inputs. As a result, existing concolic testers struggle with JavaScript, Python or Racket components whose inputs are often higher-order functions and fall back to incomplete approximations [17, 28, 31, 36].

The **goal of this paper** is to introduce provably correct foundations that lift concolic testing to the world of higher-order functions.

```
call-twice = λf. let i = f (equals 2) in
                 let j = f (equals 30) in
                 let k = f (equals 7) in
                 (cond [!(i = 12) 1]
                       [!(j = 5) 2]
                       [!(k = -2) 3]
                       [else error])
```

```
error-trigger =
  λg. (cond [(g 2) 12]
            [(g 30) 5]
            [else -2])
```

Figure 1: One Argument Call Is Not Enough; Example & Error-Triggering Input

There are three interdependent challenges for the design of a correct higher-order concolic tester. First, a higher-order concolic tester needs to be able to generate sufficiently complex function inputs to explore the behavior of a user program. Even in simple higher-order programs, this set of inputs includes functions with sophisticated structure. The left-hand side of figure 1 displays one such program, `call-twice`. It consumes a higher-order function **f** that when given a predicate on numbers returns a number. It calls **f** with three different predicates that return true if their input is `2`, `30` and `7` respectively. If the result of any of these calls is different than a specific number, `call-twice` terminates successfully; otherwise `call-twice` errors. Hence, only a fine-tuned input can make `call-twice` error. In particular, it has to be a function that calls its argument at least twice with different numbers and returns the right result in each case, like the counterexample on the right-hand side of the figure.

The second challenge is that a higher-order concolic tester needs to be able to generate structurally complex function inputs in a directed manner. Specifically, to preserve the character of first-order concolic testing, a higher-order concolic tester must start with a default input that evolves, with each run of the user program and the help of an SMT solver, to a new input that aims to exercise a previously unexplored region of the program. Returning to the example from figure 1, a higher-order concolic tester should start from a simple **f** such as a constant function and then use hints from the evaluation of the example to add appropriate calls inside **f** that call **f**'s argument, targeting the last branch of `call-twice`'s `cond` expression.

```
date< = λd1. λd2. (or ((date-year d1) < (date-year d2))
                      ((date-month d1) < (date-month d2))
                      ((date-day d1) < (date-day d2)))
main = λdates. (let sorted-dates = (sort dates date<) in ···)
```

Figure 2: Broken Argument for a Library Function.

The third challenge is that, in a higher-order setting, concretization demands that the concolic tester is ready to concretize any call to a higher-order function. For example the `main` function in figure 2 takes as input a list of dates, calls `sort` with the comparison function `date<` and expects the results to be lexicographically sorted (as there are many reasons why sorting is necessary we leave the details to the imagination of the reader). If `sort` is a library function whose implementation is inaccessible, then the concolic tester has to concretize the call to `sort` and disable symbolic evaluation for the extent of that call. Unfortunately, `date<` does not implement the lexicographical order and discovering this requires the concolic tester to track symbolically the flow of values in and out of `date<` in order to generate a list of dates that exhibits the bug. In other words, the concolic tester should be able to perform "partial" concretization so that `date<` interacts with `sort` in a concrete manner while the the evaluation of `date<` still produces the symbolic information the tester needs.

**Our paper contributes** the first formal model for a concolic tester for higher-order functions that meets all three challenges:

1. Inspired by the function application rules of unknown symbolic values in higher-order symbolic evaluation [32, 33], we construct a novel set of canonical functions that the concolic tester uses to generate inputs. We prove that if a higher-order program under test errors for some input, there is a canonical input that triggers an error too (representation completeness).
2. We devise input constraints to record at runtime facts about the structure of the generated function inputs separately from the first-order control flow path formulas from the symbolic evaluation of the user program. We specify an input evolution process that captures how the concolic tester can use input constraints to iteratively search through the space of canonical functions with the help of an SMT solver. We establish that, relative to the completeness of the solver, the concolic tester can always start with a default input and, through evolution, generate a counter-example, if one exists (search completeness). Furthermore the input evolution is directed by the input constraints that the concolic tester collects (directness).
3. Building on top of higher-order contracts [16], we develop concretization that employs wrappers around higher-order functions that are consumed by library and other inaccessible code. The wrappers allow the concolic tester to maintain control of function inputs and evaluate their bodies symbolically while producing concrete values when they interact with code that the concolic tester does not control. We prove that, in the presence of concretization, the search for the bug is not complete but the concolic tester still evaluates user programs consistently with respect to concrete evaluation (soundness).

The remainder of the paper is organized as follows. Section 2 gives an in depth by-example presentation of our approach to higher-order concolic testing. Section 3 presents our formal model and section 4 establishes its correctness properties. Section 5 describes a proof-of-concept implementation of our model that provides evidence that the model is a reasonable basis for the development of effective higher-order testers. Finally, section 6 places our results in the context of related work and section 7 offers some concluding thoughts.

## 2 Higher-order Concolic Testing by Example

The linguistic setting of our exposition of concolic testing is a small call-by-value dynamically-typed functional language without mutable state. Furthermore, we represent bugs explicitly as the term `error` and assume that user programs come with type-like input specifications.

### 2.1 First-Order Concolic Execution in a Nutshell

The goal of a concolic tester is to find a value for the inputs to a user program that cause the execution to reach `error`. To do so, the tester runs the user program in a *concolic loop* with a different input for each loop iteration. There are two differences between concolic evaluation and concrete evaluation. To explain them, consider the user program in the left-hand column of figure 3, where **X** represents the numeric input.

| | | |
|---|---|---|
| (cond<br>  [($\mathbf{X}\times\mathbf{X} - \mathbf{X} - 992 = 0$)<br>   (cond<br>    [($\mathbf{X} < 0$) error]<br>    [else 12])]<br>  [else 11]) | **Input:**<br>$\mathbf{X} \mapsto 0$<br><br>**Log:**<br>• cond: (false)<br>$\langle \mathbf{X}\times\mathbf{X} - \mathbf{X} - 992 = 0\rangle$ | **Input:**<br>$\mathbf{X} \mapsto 32$<br><br>**Log:**<br>• cond: (true)<br>$\langle \mathbf{X}\times\mathbf{X} - \mathbf{X} - 992 = 0\rangle$<br>• cond: (false)<br>$\langle \mathbf{X} < 0\rangle$ |

Figure 3: A First, First-order Concolic Example

The first difference is that, instead of concrete values, concolic evaluation utilizes values of the form $\langle \mathbf{t}\rangle$, where $\mathbf{t}$ is a first-order formula over the input variables that codifies the provenance of the value. Concretely, assume that in the first run of our example program the concolic tester picks the concrete input `0`. Instead of just starting the evaluation of the program by replacing **X** with `0`, the concolic machine keeps an environment that maps **X** to `0` and runs the program with the concolic value $\langle \mathbf{X}\rangle$ as the input. The concrete counterpart of a concolic value can be computed from the concrete values in the environment and the (first-order) formula $\mathbf{t}$ at any point during concolic evaluation.

To kick-off concolic evaluation, the concolic machine evaluates the test expression of the outer `cond` of the example. Specifically the primitive operation × detects that its input is $\langle \mathbf{X}\rangle$ and returns $\langle \mathbf{X}\times\mathbf{X}\rangle$. Even though the concrete counterparts of both of these concolic values are `0`, they bear a different relation to the input **X**. The concolic machine proceeds with the rest of the evaluation of the test expression, yielding $\langle \mathbf{X}\times\mathbf{X} - \mathbf{X} - 992 = 0\rangle$. At this point, the concolic machine uses the concrete counterpart of the concolic value and thus decides to follow the "else" branch of the outer `cond`. Hence, the first run does not trigger `error`.

The second characteristic of concolic evaluation are the connections it creates between the inputs and the evaluation of a user program. Specifically, the concolic machine logs the concolic value of the test expressions of `cond` expressions in the user program in the order they are evaluated; we refer to these entries of the log as *path constraints*. The middle column of figure 3 shows the log (and the inputs) for the run of our example when **X** is 0. Since only one `cond` expression is evaluated, the log contains a single path constraint that the concrete counterpart of the concolic value ‹**X**×**X** - **X** - 992 = 0› is false, that is the first branch of the `cond` was not taken. Intuitively, the path constraint connects the evaluation of a `cond` expression with the input to the program via concolic values. After the first run, the concolic tester asks the SMT solver for an input where **X**×**X** - **X** - 992 = 0 holds, forcing the branch to go the other way. The SMT solver may respond with 32, leading to the run represented in the right-hand column of figure 3. That run again fails to trigger the `error`, but has a log showing that the first branch of the outer `cond` was taken this time because **X**×**X** - **X** - 992 = 0 is true. It also has another constraint that indicates that the first branch of the inner `cond` was not taken because **X** < 0 is false. At this point, the concolic tester can formulate a new SMT problem that requires both **X**×**X** - **X** - 992 = 0 and **X** < 0 to be true. The problem is satisfiable and the SMT solver replies that the new concrete value for **X** should be -31, which uncovers the `error`.

### 2.2 From Numbers to Function Inputs

As described so far, concolic testing cannot handle inputs that are not numbers or other data types that SMT solvers understand. The concolic tester relies solely on a solver to generate new inputs and for that it needs to prepare a first-order problem that the solver can solve. Our first insight to surpass this restriction is to split the generation of function inputs into two subproblems:

1. testing programs with first-order function inputs and;
2. testing programs with higher-order function inputs.

As with many problems that involve higher-order functions, the first subproblem is the hard one. The solution for the second subproblem falls out of that for the first one, exploiting the natural co- and contravariance of higher-order functions. So, we first focus on first-order function inputs and we return to higher-order inputs in section 2.5.

The left-hand column in figure 4 shows a program whose input **F** is a first-order function from numbers to numbers. One of the many functions that can trigger `error` in this example is λ**x**. 2-**x**. However, a key aspect of our approach is recognizing that we care only about the behavior of the input when given 1 and 2. Since the program calls **F** with only those arguments, other arguments are irrelevant. In general, any program that terminates calls its input a finite number times so the concolic tester can model first-order function inputs as functions that look up values from a table, which we represent with a `case` expression.

As with non-function inputs, the concolic tester starts with the simplest possible function input: λ**x**. (`case` **x**), as shown in the middle column of figure 4.

```
(cond
  [((F 1) × 3 = (F 2) + 3)
   error]
  [else 11])
```

**Input:**

**F** ↦ λ**x**. (case **x**)

**Log:**

- call: (**F** 1)
- call: (**F** 2)
- cond: (false) ‹0×3 = 0+3›

**Input:**

**F** ↦ λ**x**.(case **x** [1 ‹**Y**›] [2 ‹**Z**›])

**Y** ↦ 0

**Z** ↦ 0

**Log:**

- call: (**F** 1)
- call: (**F** 2)
- cond: (false) ‹**Y**×3 = **Z**+3›

Figure 4: First-order Input

This function looks up its argument in an empty table and returns always 0. If the concolic machine treated this function as a first-order input, it would record that the first branch of cond was not taken because ‹(**F** 1) × 3 = (**F** 2) + 3› is false. This formula, however, involves function symbols which SMT solvers cannot handle when higher-order functions come into play. Thus the concolic machine does not record the constraint and instead simply reduces all applications of **F** en route to the concolic value of the test expression. Unfortunately, this first function input does not help the concolic tester make progress. Since the input returns the constant 0 for any argument, the concolic value of the test loses any connection to **F** and the concolic tester does not have much leverage to adjust **F**'s behavior and affect the evaluation of the program.

To rectify the situation our concolic tester aims to generate a new input with a shape that gives to the tester increased control over **F**'s behavior. The pivotal idea that enables the input evolution process is that the concolic machine logs so called *input constraints*. That is, in addition to the path constraints of the user program, it also records the values that the user program provides to **F**, or any other function input. Back to the example, the evaluation records two input constraints: one for argument 1 and one for 2. The middle column of figure 4 shows the new log entries along with the path constraint from the evaluation of the cond expression.

With the input constraint from the log, the concolic tester can construct a second function input as shown in the right-hand column of figure 4. This new function input has a case expression with two clauses: one for when the argument is 1 and one for when it is 2. Furthermore the concolic tester introduces two fresh input variables **Y** and **Z** as the actions of the two clauses. The initial values for these two new inputs are both 0. However, exactly because the results of the function are input variables rather than mere constants, the concolic tester can configure the values for these inputs to trigger the error with the help of an SMT solver. Specifically, the concolic value of the test of the first branch of the cond expression in the example becomes ‹**Y**×3 = **Z**+3›, as shown in the log. This problem has solutions and the SMT solver discovers that **Y**=1 and **Z**=0 are sufficient to "switch" the evaluation of the conditional, which triggers the error.

In sum, to handle first-order function inputs, the concolic tester starts with the simplest possible function, records input constraints that describe the arguments that the function consumes, uses the constraints to generate a new function that, in turn, introduces fresh inputs, and finally employs the SMT solver to fine-tune the values for these inputs.

As a final remark in this section, function inputs are regular functions that behave like a concrete input would behave. For the concolic machine though, the evaluation of their bodies is a source of new information that powers the subsequent iterations of the concolic loop. This is a key observation for concretization in the our setting. A concolic tester concretizes calls to functions when it cannot evaluate their bodies in a concolic manner. This situation arises when the function comes from an external library, such as `sort` from section 1, and the function's code is not under the control of the concolic machine. In the context of this section, this translates to the situation where the function's body cannot interact with any concolic values nor can its evaluation record path constraints in the log of the machine. A naive solution to the issue is that the concolic machine computes the concrete counterpart of the argument, delegates the call of the function to a concrete machine and then uses the result of the concrete call to proceed. This means, however, that the concolic machine loses any constraints from the evaluation of the body of the argument if the argument is a function itself. Instead, our concolic machine uses a proxy argument for the concrete call that wraps the actual argument. Thus calls to the argument go back to the concolic machine that records all the usual constraints and only concretizes any first-order results the argument produces. We return to our approach to concretization in section 3.3.

### 2.3 Input Interactions

The previous example supplies a constant number to **F**. However, programs can also supply other, first-order inputs to their function inputs, as in the example in the left-hand column of figure 5.

| | | |
|---|---|---|
| (cond<br>[((**F** **X**) × 3 = (**F** (**X**×2)) + 3)<br>error]<br>[else 11]) | **Input:**<br>**F** ↦ λ**x**. (case **x**)<br>**X** ↦ 0<br><br>**Log:**<br>• call: (**F** ‹**X**›)<br>• call: (**F** ‹**X**×2›)<br>• cond: (false) ‹0×3 = 0+3› | **Input:**<br>**F** ↦ λ**x**.(case **x** [‹**X**› ‹**Y**›] [‹**X**×2› ‹**Z**›])<br>**X** ↦ 1<br>**Y** ↦ 0<br>**Z** ↦ 0<br><br>**Log:**<br>• call: (**F** ‹**X**›)<br>• call: (**F** ‹**X**×2›)<br>• cond: (false) ‹**Y**×3 = **Z**+3› |

Figure 5: Interacting Inputs

In order to trigger the error in this example, **F** must be able to return different results from its two different calls. However, if the initial concrete value for **X** is 0, the concrete counterparts of the arguments to the calls to **F** are the same for both calls. Thus, if the concolic machine logs only the concrete counterparts of the arguments as part of input constraints, the concolic tester loses the connection between **X** and the values that the user program passes to **F**. Instead, the concolic machine uses the concolic values when logging input constraints. As shown in the log in the middle column of figure 5, the concolic values of the arguments to the two calls to **F** are ‹**X**› and ‹**X**×2›. Thus, the concolic tester can extend the case of **F** with two clauses, one for when the concrete counterpart of the argument of **F** matches that of ‹**X**› and one when it matches that of ‹**X**×2›. The effect of this extension is that any problems the concolic tester sends to the SMT solver contain the additional constraint that **X** and **X**×2 are different. Consequently, in a manner similar to the previous example, the concolic tester eventually uses 1 as the concrete value for **X** and discovers the error. The right-hand column of figure 5 displays this counter-example.

### 2.4 Blind Extensions Are Not Enough

So far we have seen how the concolic tester uses input constraints and concolic values to extend the case expression of a first-order function input. However, the extension may lead the concolic tester to a dead-end. This is a subtle point that, unfortunately, requires a complex example to illustrate. Figure 6 contains the simplest one we know.

This example is complex enough that it deserves a brief walkthrough. To start, note that it has two inputs, **F**, a function from numbers to numbers, and **X**, a number, and that reaching the error requires that the tests of all of the branches of the cond expression of the example fail. In effect, the condition for triggering error is the conjunction of the four formulas that follow the negations in the example. To confirm that this example does have a error-triggering input, take **X** to be -10 and **F** to be λ**x**. 11 × (**x**+11).

If the concolic tester follows the process described so far in this section, it manages to generate an input that makes the tests of the first three branches of cond to fail. But then, it seems impossible for the concolic tester to extend the input further to make the test of the fourth branch cond succeed. To see how this plays out, the middle column in figure 6 shows the state of the concolic machine after a few iterations of the concolic loop. The concolic tester first runs the example with the default constant zero function as the input, which results in 11 and logs the argument **X** for **F**; the concolic tester then extends the case of **F** with a clause that returns a fresh concolic variable **Y**. It then discovers **Y** must be set to 11 to skip the first branch in the cond expression. For this input, the example produces result 7, failing to also skip the second branch of the cond expression. After another iteration, the concolic tester manages to skip the second branch of cond and generates the input shown in the middle column of figure 6.

```
(cond
  [!(F X = 11) 1]
  [!(F 0 = 121) 7]
  [!(F (X+10) = 121) 2]
  [!(X = -10) 9]
  [else error])
```

**Input:**

**F** ↦
λ**x**.(case **x**
  [‹**X**› ‹**Y**›]
  [0 ‹**Z**›])

**X** ↦ 1
**Z** ↦ 121
**Y** ↦ 11

**Log:**

- call: (**F** ‹**X**›)
- cond: (false) ‹!(**Y** = 11)›
- call: (**F** 0)
- cond: (false) ‹!(**Z** = 121)›
- call: (**F** ‹**X**+10›)
- cond: (true) ‹!(0 = 121)›

**Input:**

**F** ↦
λ**x**.(case **x**
  [‹**X**› ‹**Y**›]
  [0 ‹**Z**›]
  [‹**X**+10› ‹**W**›])

**X** ↦ 1
**W** ↦ 121
**Z** ↦ 121
**Y** ↦ 11

**Log:**

- call: (**F** ‹**X**›)
- cond: (false) ‹!(**Y** = 11)›
- call: (**F** 0)
- cond: (false) ‹!(**Z** = 121)›
- call: (**F** ‹**X**+10›)
- cond: (false) ‹!(**W** = 121)›
- cond: (true) ‹!(**X** = -10)›

Figure 6: A Complex, Subtle Example

Let us analyze the middle section of the figure to understand the concolic tester's state at this point in the process. The input consists of a function **F** that returns ‹**Y**› when it sees the input ‹**X**›, where **Y** is 11 and returns ‹**Z**› when it sees 0, where **Z** is 121. When we feed this input to the program in the left-hand column, we skip the first and second branches of the cond, because **F** has been tuned to get through them. This part of the execution produces the first four entries in the log. Next the concolic machine arrives at the third branch of the cond and the call (**F** (**X**+10)), which produces the fifth entry in the log. The concrete value of the argument is 11, which has no matching clause in the case of **F** so **F** returns 0, and the program terminates with 2, following the fourth branch as recorded in the last entry in the log.

The straightforward next step is to insist that this third call has its own distinct clause in **F**, meaning the concolic engine asks the solver for a solution to the equations !(**X** = 0) and !(0 = **X**+10). An input based on the solution to these equations is shown in the third column of figure 6, and it too deserves a careful look. The log is identical up to the last "call" entry so the program evaluates the same to that point. The next entry in the log (second to last) reveals the concolic machine skips the third branch of the cond and thus proceeds with the evaluation of the test **X** = -10 of the fourth branch. Since the value for the input **X** is 1, the machine follows the branch and the program returns 9.

Clearly, since we want the machine to skip the fourth branch too, the tester should present to the solver the same set of equations that lead to the latest input and assert in addition **X** = -10. Unfortunately, there is no solution to these equations since they already contain `!(X=-10)` because the first and third clauses of **F** are distinct.

While it is usually a good choice for the concolic tester to force the arguments the user program provides to function inputs to be distinct, in some cases, like this one, it is necessary to do otherwise. Indeed, at the very point of this example to be able to reach `error`, we need to improve the concolic tester's capabilities. More precisely, the concolic tester needs to be able to take a new argument and force it into an existing clause rather than adding a new one. In this example, if the concolic tester forces the argument `X+10` and the argument `0` to match the same clause, then it can add the equation `0=X+10` to the problem it presents to the SMT solver at the end of the iteration of the concolic loop described in the middle column of figure 6. This extra equation no longer clashes with the necessary equation to skip the fourth branch of the user program (`!(X=-10)`) and with the help of the SMT solver, the tester can adjust the input in the middle column of figure 6 to use `-10` as **X** and trigger the `error`.

To sum up, at the end of each iteration of the concolic loop there are multiple ways a first-order input can evolve. The concolic tester can use the logged input constraints to assert to the SMT solver that the arguments of a call to the input are different from those of some other calls and extend the `case` expression of the input accordingly (section 2.2 to section 2.3). Or, it can assert to the SMT solver that the arguments of two calls to the input are equal (section 2.4). In either case, the concolic tester asks the SMT solver to determine the values of first-order inputs. We revisit formally the evolution of inputs in section 3.2. As a concluding note, we underline that the concolic tester may have to try any number of the possible ways an input can evolve. The strategy the concolic tester uses to prioritize and search the space of these possibilities is out of the scope of this paper. Herein, we focus instead on what the concolic tester can do at each point in the concolic loop and whether a sequence of its choices is guaranteed to reveal a possible `error` in a user program.

#### 2.5 Higher-order Inputs

Handling higher-order inputs, that is functions that consume and/or return other functions, not just numbers, requires a generalization of the ideas in the previous section. However, the seed of the key insight is already there in the way our concolic tester handles first-order function inputs. Intuitively, the tester treats a first-order function input as a source of new, latent inputs that the concolic tester provides to the user program. As we discuss above this is exactly the rationale for the fresh input variables that appear in the actions of the `case` expressions of first-order function inputs.

Contravariantly, when an input consumes a function argument, the tester can simply treat the function argument as a source of further, latent arguments that the user program provides. The input can decide how and when to call its function argument in order to obtain these latent arguments. These function calls, in turn, open up new points where the concolic tester supplies additional inputs to the user program.

| | | |
|---|---|---|
| (cond<br>[(**G** (λ**x**. **x**+1) + **G** (λ**x**. **x**+2) = 9)<br>error]<br>[else 11]) | **Input:**<br>**G** ↦ λ**f**. ‹**X**› | **Input:**<br>**G** ↦ λ**f**. let **fY** = **f** ‹**Y**› in<br>(case **fY** [‹**Y**+1› ‹**X**›]<br>[‹**Y**+2› ‹**Z**›]) |

Figure 7: Co- & Contravariance at Work

Concretely, consider the left-hand program in figure 7. It has one input, **G**, which consumes a function **f** on numbers and returns a number. As before, the concolic tester starts out by generating the constant zero function. Of course, this does not uncover the `error` so, same as for first-order function inputs, the concolic tester turns to the input constraints in its log. However, the log simply shows that the user program provides **G** with two procedures. Therefore the `case`-expression approach does not apply in a straightforward manner. The concolic tester can change the input **G** to return a fresh input variable **X** as in the middle column of figure 7. Unfortunately, this still does not help trigger the `error`.

While many programming languages offer a certain notion of physical equality for procedures, our approach is for the concolic tester to generate a function **G** that calls its argument **f** and then inspects the result **fY** with a `case` expression as if it was yet another argument to **G**. In this case, **G** calls **f** with a fresh input variable **Y** then binds the result to **fY** which acts as a latent argument that the user program provides to **G**. To account for latent arguments, we generalize input constraints to keep track of variables such as **fY** together with the results of calls to function arguments.

The overall effect is that the concolic tester acquires the vantage point it needs to follow the same process as for first-order function inputs. In particular, the input constraints for **fY** contain the results from calling **f** that in turn are tied to input variable **Y** and thus under the control of the concolic tester. Furthermore, just like for first-order functions, they provide guidance for filling in the clauses of the `case` expression of **G**. Concretely in our example, the input constraints for **fY** record that it is equal to either ‹**Y**+1› or ‹**Y**+2›, which the concolic tester can consider as distinct and, with the help of the SMT solver, generate the **G** on the right-hand side of figure 7 that triggers the `error`, where **X** and **Z** are fresh input variables mapped to `4` and `5` respectively.

Overall, the concolic tester handles function inputs by decomposing them one layer at a time until it ends up with first-order functions. At each point of decomposition, that is when an input calls one of its arguments, the concolic tester introduces fresh input variables and logs input constraints that connect the fresh input variables and the calls' results. Then it keeps track of these connections with input constraints and uses the constraints to fill in the `case` expressions in the bodies of higher-order function inputs. Effectively, this approach entails that the concolic tester considers inputs in a so called canonical form only. Informally, canonical inputs nest `let`-expressions and `case`-expressions. The precise definition of canonical functions and their evolution are the subject of Section 3 along with the rest of the model for higher-order concolic testing.

## 3 Formalizing Higher-order Concolic Testing

The core of our formal model of higher-order concolic testing is a concolic (abstract) machine that loads and runs user programs, and the input evolution metafunction that generates inputs for the next run.

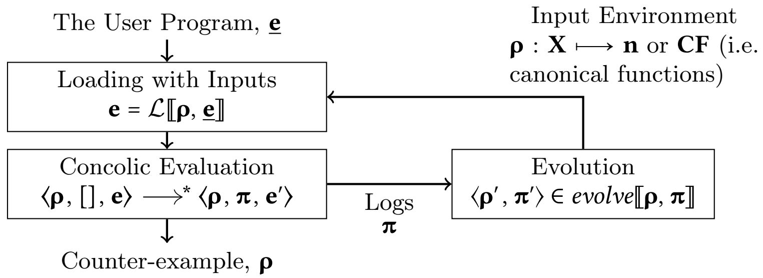

Figure 8: The Full Input Evolution Cycle

Figure 8 depicts how the concolic machine and the input evolution metafunction work together to form the concolic loop. At the beginning of each iteration of the loop, the load metafunction $\mathcal{L}$ consumes the environment $\boldsymbol{\rho}$ that maps each input variable $\mathbf{X}$ in the user program $\underline{\mathbf{e}}$ to a value and prepares the user program for the concolic machine. The concolic machine evaluates the loaded program, $\mathbf{e}$, with the help of two registers: the environment of inputs $\boldsymbol{\rho}$ and the log $\boldsymbol{\pi}$ (that is initially empty). If the result of the evaluation is not an error, the final content of log $\boldsymbol{\pi}$ together with the environment $\boldsymbol{\rho}$ determine how the input evolves. Specifically, the *evolve* metafunction uses them to compute a list of pairs that each contains a new environment of inputs $\boldsymbol{\rho}'$ and a prediction of the contents of the log $\boldsymbol{\pi}'$ of the concolic machine after evaluating the program with $\boldsymbol{\rho}'$. The concolic loop repeats and, with each iteration, explores one more input. When it discovers an error in the user program, the loop terminates and the environment of the error-generating input turns into a concrete counter-example.

Section 3.1 details the concolic machine, section 3.2 formalizes the evolution function and section 3.3 extends the model with concretization.

### 3.1 From User Programs to Concolic Evaluation

$\mathbf{op}$ ::= `!` | `+` | `-` | `×` | `<` | `=` | `integer?` | `procedure?`
$\underline{\mathbf{e}}$ ::= $\mathbf{n}$ | `error` | $\mathbf{x}$ | $\mathbf{X}$ | $(\lambda\mathbf{x}.\ \underline{\mathbf{e}})$ | $\mathbf{op}\ \underline{\mathbf{e}}$ | $\mathbf{op}\ \underline{\mathbf{e}}\ \underline{\mathbf{e}}$ | $\underline{\mathbf{e}}\ \underline{\mathbf{e}}$ | (`cond` [$\underline{\mathbf{e}}\ \underline{\mathbf{e}}$] ... [`else` $\underline{\mathbf{e}}$])
$\mathbf{X}$, $\mathbf{Y}$, $\mathbf{Z}$, $\mathbf{F}$, $\mathbf{G}$, etc., are concolic variables.

Figure 9: The Syntax of User Programs

$$\begin{aligned}
\mathbf{CF} &::= (\lambda \mathbf{x}.\ \mathbf{case_x}) \\
\mathbf{case_x} &::= (\mathtt{case}^{\ell}\ \mathbf{x}) \mid (\mathtt{case}^{\ell}\ \mathbf{x}\ [\mathtt{procedure?}\ \mathbf{e}^{\circ}]^{\ell}\ [\langle \mathbf{t} \rangle\ \mathbf{e}^{\circ}]^{\ell} \ldots) \\
\mathbf{e}^{\circ} &::= \mathbf{v}^{\circ} \mid (\mathtt{let}\ \mathbf{z} = \mathbf{f}\ \mathbf{v}^{\circ}\ \mathtt{in}\ \mathbf{case_z}) \\
\mathbf{v}^{\circ} &::= \mathbf{x} \mid \langle \mathbf{X} \rangle \mid \mathbf{CF}
\end{aligned}$$

Figure 10: Canonical Functions

Figure 9 collects the constructs of the language of user programs, including numbers **n**, error, primitive operators **op** $\underline{\mathbf{e}}$ ..., multi-way conditional expressions cond, and uppercase variables **X**, **Y**, **F**, etc., for the inputs of a user program. These inputs are either numbers or, as we discuss briefly in section 2.5, functions in canonical form. The error construct represents actual bugs in user programs; dynamic type errors manifest themselves as stuck terms.

Figure 10 provides the formal definition of canonical functions. The body of a canonical function with argument **x** is a $\mathbf{case_x}$ expression with zero or more clauses. As we mention in section 2, a $\mathbf{case_x}$ that has no clauses is equivalent to the constant 0. Different than the presentation in section 2 and due to the dynamically-typed nature of our model, the very first clause of every non-empty $\mathbf{case_x}$ always checks whether **x** is a function. If **x** is a function **f**, similar to the discussion in section 2.5, the action $\mathbf{e}^{\circ}$ of the procedure? clause is typically a let expression that applies **f** and inspects the result of the application **z** with yet another case expression.[1] If **x** is a number then the $\mathbf{case_x}$ compares **x** with each of the concolic values ‹**t**› and delegates to the corresponding action $\mathbf{e}^{\circ}$. Similar to the examples of section 2, the argument $\mathbf{v}^{\circ}$ for **f** in a let expression is an input, i.e., a concolic value ‹**X**› where **X** is a fresh concolic variable, or a canonical function. Some goes for the actions $\mathbf{e}^{\circ}$ of a non-procedure? clause of a case expression. However, in these positions the model can also use variables in scope in an attempt to identify a counter-example for a user program with fewer concolic loop iterations, which is helpful when proving the metatheoretical properties of the model. In general, despite their restricted shape, canonical functions can simulate any function input that triggers an error in a user program. We return to this point in section 4.

As a final remark on canonical functions, one important difference from the discussion of function inputs in section 2.5 is that, herein, each case expression comes with labels $\ell$. There are two kinds of labels: labels that uniquely identify a case expression and labels that uniquely identify a clause of a case. As we explain further on, their purpose is to allow the concolic tester to analyze the log of the concolic machine after each iteration of the concolic loop to direct the evolution of a canonical function.

Figure 11 shows the complete definition of the concolic machine. As we mention at the beginning of this section, the machine has three registers: the input environment $\boldsymbol{\rho}$ that maps concolic variables **X** to either numbers or canonical functions; the log of constraints $\boldsymbol{\pi}$; and the term **e** the machine evaluates.

[1] We use let $\mathbf{x} = \mathbf{e}_1$ in $\mathbf{e}_2$ as shorthand for $(\lambda \mathbf{x}.\ \mathbf{e}_2)\ \mathbf{e}_1$.

$\mathbf{M} ::= \langle \boldsymbol{\rho}, \boldsymbol{\pi}, \mathbf{e} \rangle$

$\boldsymbol{\rho} : \mathbf{X} \longmapsto \mathbf{n}$ or $\mathbf{CF}$

$\boldsymbol{\pi} ::= [\mathbf{p}, ...]$

$\mathbf{p} ::= \langle$“R-COND”, “FALSE”, ‹**t**›$\rangle$ | $\langle$“R-COND”, “TRUE”, ‹**t**›$\rangle$
 | $\langle$“R-CASE”, $\boldsymbol{\ell}$, **v**, “MISS”$\rangle$ | $\langle$“R-CASE”, $\boldsymbol{\ell}$, **v**, “HIT”: $\boldsymbol{\ell}\rangle$

**t** ::= **X** | **n** | !**t** | **op t t**

**e** ::= ‹**t**› | `error` | **x** | (λ**x**. **e**) | **op e** | **op e e** | **e e** | (`cond` [**e e**] ... [`else` **e**])
 | (`case`$^{\ell}$ **e**) | (`case`$^{\ell}$ **e** [`procedure?` **e**]$^{\ell}$ [‹**t**› **e**]$^{\ell}$ ...)

**v** ::= (λ**x**. **e**) | ‹**t**›

**E** ::= []
 | **op E** | **op E e** | **op v E** | **E e** | **v E** | (`cond` [**E e**] [**e e**] ... [`else` **e**])
 | (`case`$^{\ell}$ **E**) | (`case`$^{\ell}$ **E** [`procedure?` **e**]$^{\ell}$ [‹**t**› **e**]$^{\ell}$ ...)

$\mathcal{L} : \boldsymbol{\rho}\ \underline{\mathbf{e}} \longrightarrow \mathbf{e}$ (interesting cases)

$\mathcal{L}[\![\boldsymbol{\rho}, \mathbf{n}]\!] = \langle \mathbf{n} \rangle$

$\mathcal{L}[\![\boldsymbol{\rho}, \mathbf{X}]\!] = \langle \mathbf{X} \rangle$
 where $\boldsymbol{\rho}(\mathbf{X}) = \mathbf{n}$

$\mathcal{L}[\![\boldsymbol{\rho}, \mathbf{F}]\!] = \lambda\mathbf{x}.\ \mathbf{case_x}$
 where $\boldsymbol{\rho}(\mathbf{F}) = (\lambda\mathbf{x}.\ \mathbf{case_x})$

$\mathcal{E} : \boldsymbol{\rho}\ \mathbf{t} \longrightarrow \mathbf{n}$

$\mathcal{E}[\![\boldsymbol{\rho}, \mathbf{n}]\!] = \mathbf{n}$

$\mathcal{E}[\![\boldsymbol{\rho}, \mathbf{X}]\!] = \mathbf{n}$
 where $\boldsymbol{\rho}(\mathbf{X}) = \mathbf{n}$

$\mathcal{E}[\![\boldsymbol{\rho}, \mathbf{op}\ \mathbf{t}_1 ...]\!] = \mathbf{n}$
 where $\mathbf{op} \in \{!, +, -, \times, <, =\}$,
 $\delta[\![\mathbf{op}, \mathcal{E}[\![\boldsymbol{\rho}, \mathbf{t}_1]\!], ...]\!] = \mathbf{n}$

Figure 11: The Concolic Machine and the Evaluation Language

Evaluation terms **e** are user program terms extended with canonical functions and concolic values ‹**t**›. Recall from section 2 that the latter keep track of the provenance of a value as a symbolic first-order formula **t** that an SMT solver can handle. The concrete counterpart of a concolic value can be computed at any point in the evaluation from **t** and the input environment $\boldsymbol{\rho}$ of the concolic machine with the simple $\mathcal{E}$ metafunction.

The log, $\boldsymbol{\pi}$, of the concolic machine collects two kinds of constraints, **p**. Path constraints are either $\langle$“R-COND”, “FALSE”, ‹**t**›$\rangle$ or $\langle$“R-COND”, “TRUE”, ‹**t**›$\rangle$ and are logged by evaluating `cond` expressions. The first indicates that the test of a branch failed during concolic evaluation; the second that the test succeeded. In either case, the concolic value of the test is ‹**t**› where the symbolic first-order formula **t** codifies the necessary and sufficient condition for the test to succeed.

Input constraints, $\langle$“R-CASE”, $\boldsymbol{\ell}$, **v**, “HIT”: $\boldsymbol{\ell}_i\rangle$ and $\langle$“R-CASE”, $\boldsymbol{\ell}$, **v**, “MISS”$\rangle$, are logged by evaluating `case` expressions in canonical functions. The label $\boldsymbol{\ell}$ associates each input constraint with a `case` expression in the input environment $\boldsymbol{\rho}$. A $\langle$“R-CASE”, $\boldsymbol{\ell}$, **v**, “HIT”: $\boldsymbol{\ell}_i\rangle$ constraint indicates that the `case` expression with label $\boldsymbol{\ell}$ given value **v** followed the action of its clause with label $\boldsymbol{\ell}_i$. A $\langle$“R-CASE”, $\boldsymbol{\ell}$, **v**, “MISS”$\rangle$ indicates that the `case` with label $\boldsymbol{\ell}$ given value **v** followed the implicit in our model “else” clause, whose action is the constant `0`. Since the first thing a canonical function does when it interacts with the user program is to inspect the value it receives with `case`, some of the values **v** in input constraints are exactly the values that the user program provides to function inputs and consequently the concolic tester. Others are the results of calls to functions of the user program that higher-order function inputs perform with their `let` expressions, which are also values that the user program provides to the concolic tester as we discuss in section 2.5. Hence the input constraints here supersede the simplified input constraints from section 2.

Since concolic evaluation handles concolic rather than concrete values, the $\mathcal{L}[\![\boldsymbol{\rho}, \underline{\mathbf{e}}]\!]$ metafunction prepares a user program $\underline{\mathbf{e}}$ accordingly for the concolic machine. It traverses $\underline{\mathbf{e}}$ and replaces every integer **n** with ‹**n**›, concolic variables **X** with ‹**X**› if $\boldsymbol{\rho}$ maps **X** to an integer and **F** with the actual function if $\boldsymbol{\rho}$ maps **F** to a canonical function. Note that $\mathcal{L}$ does not introduce any ‹**F**› since $\boldsymbol{\rho}(\mathbf{F})$ can be a higher-order function which, in general, SMT solvers have no theory for.

Given a loaded program, the concolic machine operates in accordance with the reduction rules from figure 12. The rules can be divided into four groups. Group Sym implements base-value provenance tracking for primitive operators. For primitive operators that have straightforward SMT formula counterparts, rule [R-Trace1] produces a concolic value whose formula is formed by the operator and the symbolic provenance of the operands. Otherwise, [R-Trace2] discards the provenance information of the operands and simply returns the concolic value ‹**n**› where **n** is the concrete result of the operation.

The next group, Cond, includes the rules for `cond` expressions. In general, the concolic machine inspects the concrete counterpart of the value of the test expression in the first clause of a `cond` determine whether to take or skip a branch. When $\mathcal{E}[\![\boldsymbol{\rho}, \mathbf{t}]\!]$ is non-zero, [R-CondTrue] proceeds with the action expression $\mathbf{e}_1$ of the first clause and logs the path constraint ⟨"R-Cond", "True", ‹**t**›⟩. When $\mathcal{E}[\![\boldsymbol{\rho}, \mathbf{t}]\!]$ is zero, rule [R-CondFalse] drops the first clause of the `cond` and appends the path constraint ⟨"R-Cond", "False", ‹**t**›⟩ to the list of path constraints. If `cond` has no other clauses but the `else` one, [R-CondElse] replaces the conditional expression with the action expression `e` of its `else` clause.

The third group, Case, describe the evaluation of `case` expressions from canonical functions. When evaluating a `case` expression, the concolic machine searches the clauses for a match. If the `case` expression is empty or if the input (**v**) is a concolic value whose concrete counterpart is a number that is different from tests of all clauses, [R-CaseMiss1] and [R-CaseMiss2] (respectively) reduce the `case` expression to the default action expression ‹0›. They also append the input constraint ⟨"R-Case", $\boldsymbol{\ell}$, **v**, "Miss"⟩ to the log. Otherwise, the last two rules of the group handle successful matches. For cases where the input **v** is a function λ**x**. **e**, [R-CaseHit1] reduces `case` to the action expression of its first clause `e`. For cases where the input **v** is a concolic value ‹**t**›, rule [R-CaseHit2] selects the matching clause with label $\boldsymbol{\ell}_i$ and reduces `case` to the corresponding action $\mathbf{e}_i$. Both rules log the input constraint ⟨"R-Case", $\boldsymbol{\ell}$, **v**, "Hit": $\boldsymbol{\ell}_i$⟩ with the label $\boldsymbol{\ell}$ of the `case` expression, the input **v** and the label $\boldsymbol{\ell}_i$ of the matching clause.

The last group, Other, completes the definition of the reduction rules. Rule [R-App] is the standard call-by-value $\beta$-reduction while rule [R-Error] and [R-Ctxt] close the rules over evaluation contexts.

**Group Sym**

$$\frac{\mathbf{op} \in \{!, +, -, \times, <, =\}}{\langle \rho, \pi, \mathbf{op}\ \langle \mathbf{t}_1 \rangle \ldots \rangle \longrightarrow \langle \rho, \pi, \langle \mathbf{op}\ \mathbf{t}_1 \ldots \rangle \rangle}\ [\text{R-Trace1}]$$

$$\frac{\mathbf{op} \in \{\texttt{integer?}, \texttt{procedure?}\}}{\langle \rho, \pi, \mathbf{op}\ \mathbf{v} \rangle \longrightarrow \langle \rho, \pi, \langle tag[\![\mathbf{op}, \mathbf{v}]\!] \rangle \rangle}\ [\text{R-Trace2}]$$

$$\begin{aligned}
&tag : \mathbf{op}\ \mathbf{v} \longrightarrow 0 \text{ or } 1 \\
&tag[\![\texttt{integer?}, \langle \mathbf{t} \rangle]\!] &&= 1 \\
&tag[\![\texttt{integer?}, (\lambda \mathbf{x}.\ \mathbf{e})]\!] &&= 0 \\
&tag[\![\texttt{procedure?}, \langle \mathbf{t} \rangle]\!] &&= 0 \\
&tag[\![\texttt{procedure?}, (\lambda \mathbf{x}.\ \mathbf{e})]\!] &&= 1
\end{aligned}$$

**Group Cond**

$$\frac{\mathcal{E}[\![\rho, \mathbf{t}]\!] \neq 0 \qquad \pi' = \pi +\!\!+ [\langle \text{“R-Cond”}, \text{“True”}, \langle \mathbf{t} \rangle \rangle]}{\langle \rho, \pi, (\texttt{cond}\ [\langle \mathbf{t} \rangle\ \mathbf{e}'_1]\ [\mathbf{e}_2\ \mathbf{e}'_2] \ldots [\texttt{else}\ \mathbf{e}'_k]) \rangle \longrightarrow \langle \rho, \pi', \mathbf{e}'_1 \rangle}\ [\text{R-CondTrue}]$$

$$\frac{\mathcal{E}[\![\rho, \mathbf{t}]\!] = 0 \qquad \pi' = \pi +\!\!+ [\langle \text{“R-Cond”}, \text{“False”}, \langle \mathbf{t} \rangle \rangle]}{\begin{array}{l}\langle \rho, \pi, (\texttt{cond}\ [\langle \mathbf{t} \rangle\ \mathbf{e}'_1]\ [\mathbf{e}_2\ \mathbf{e}'_2] \ldots [\texttt{else}\ \mathbf{e}'_k]) \rangle \longrightarrow \\ \langle \rho, \pi', (\texttt{cond}\ [\mathbf{e}_2\ \mathbf{e}'_2] \ldots [\texttt{else}\ \mathbf{e}'_k]) \rangle\end{array}}\ [\text{R-CondFalse}]$$

$$\frac{}{\langle \rho, \pi, (\texttt{cond}\ [\texttt{else}\ \mathbf{e}]) \rangle \longrightarrow \langle \rho, \pi, \mathbf{e} \rangle}\ [\text{R-CondElse}]$$

**Group Case**

$$\frac{\pi' = \pi +\!\!+ [\langle \text{“R-Case”}, \ell, \mathbf{v}, \text{“Miss”} \rangle]}{\langle \rho, \pi, (\texttt{case}^{\ell}\ \mathbf{v}) \rangle \longrightarrow \langle \rho, \pi', \langle 0 \rangle \rangle}\ [\text{R-CaseMiss1}]$$

$$\frac{\mathcal{E}[\![\rho, \mathbf{t}]\!] \notin \{\mathcal{E}[\![\rho, \mathbf{t}_2]\!], \ldots\} \qquad \pi' = \pi +\!\!+ [\langle \text{“R-Case”}, \ell, \langle \mathbf{t} \rangle, \text{“Miss”} \rangle]}{\langle \rho, \pi, (\texttt{case}^{\ell}\ \langle \mathbf{t} \rangle\ [\texttt{procedure?}\ \mathbf{e}_1]^{\ell_1}\ [\langle \mathbf{t}_2 \rangle\ \mathbf{e}_2]^{\ell_2} \ldots) \rangle \longrightarrow \langle \rho, \pi', \langle 0 \rangle \rangle}\ [\text{R-CaseMiss2}]$$

$$\frac{\pi' = \pi +\!\!+ [\langle \text{“R-Case”}, \ell, (\lambda \mathbf{x}.\ \mathbf{e}), \text{“Hit”}: \ell_1 \rangle]}{\langle \rho, \pi, (\texttt{case}^{\ell}\ (\lambda \mathbf{x}.\ \mathbf{e})\ [\texttt{procedure?}\ \mathbf{e}_1]^{\ell_1}\ [\langle \mathbf{t}_2 \rangle\ \mathbf{e}_2]^{\ell_2} \ldots) \rangle \longrightarrow \langle \rho, \pi', \mathbf{e}_1 \rangle}\ [\text{R-CaseHit1}]$$

$$\frac{\begin{array}{c}[\langle \ell_2, \mathbf{t}_2, \mathbf{e}_2 \rangle, \ldots] = [\langle \ell_p, \mathbf{t}_p, \mathbf{e}_p \rangle, \ldots] +\!\!+ [\langle \ell_i, \mathbf{t}_i, \mathbf{e}_i \rangle] +\!\!+ [\langle \ell_s, \mathbf{t}_s, \mathbf{e}_s \rangle, \ldots] \\ \mathcal{E}[\![\rho, \mathbf{t}]\!] \notin \{\mathcal{E}[\![\rho, \mathbf{t}_p]\!], \ldots\} \qquad \mathcal{E}[\![\rho, \mathbf{t}]\!] = \mathcal{E}[\![\rho, \mathbf{t}_i]\!] \\ \pi' = \pi +\!\!+ [\langle \text{“R-Case”}, \ell, \langle \mathbf{t} \rangle, \text{“Hit”}: \ell_i \rangle]\end{array}}{\langle \rho, \pi, (\texttt{case}^{\ell}\ \langle \mathbf{t} \rangle\ [\texttt{procedure?}\ \mathbf{e}_1]^{\ell_1}\ [\langle \mathbf{t}_2 \rangle\ \mathbf{e}_2]^{\ell_2} \ldots) \rangle \longrightarrow \langle \rho, \pi', \mathbf{e}_i \rangle}\ [\text{R-CaseHit2}]$$

**Group Other**

$$\frac{}{\langle \rho, \pi, (\lambda \mathbf{x}.\ \mathbf{e})\ \mathbf{v} \rangle \longrightarrow \langle \rho, \pi, \mathbf{e}\{\mathbf{x} \mapsto \mathbf{v}\} \rangle}\ [\text{R-App}]$$

$$\frac{\langle \rho, \pi_1, \mathbf{e}_1 \rangle \longrightarrow \langle \rho, \pi_2, \mathbf{e}_2 \rangle}{\begin{array}{l}\langle \rho, \pi_1, \mathbf{E}[\mathbf{e}_1] \rangle \longrightarrow \\ \langle \rho, \pi_2, \mathbf{E}[\mathbf{e}_2] \rangle\end{array}}\ [\text{R-Ctxt}] \qquad \frac{\mathbf{E} \neq [\,]}{\begin{array}{l}\langle \rho, \pi, \mathbf{E}[\texttt{error}] \rangle \longrightarrow \\ \langle \rho, \pi, \texttt{error} \rangle\end{array}}\ [\text{R-Error}]$$

Figure 12: The Reduction Relation of Concolic Evaluation

Before concluding, it is worth mentioning that if the concolic evaluation of a user program raises `error`, it is straightforward for the concolic tester to produce a counter-example in the language of user programs. All the necessary information is in the latest input environment of the concolic machine.

#### 3.2 Evolution of Higher-order Inputs

If the concolic machine evaluates a user program without raising an error, the metafunction $\mathit{evolve}[\![\boldsymbol{\rho}, \boldsymbol{\pi}]\!]$ analyzes the log of the machine and compiles a list of new input environments. Specifically, for each constraint from $\boldsymbol{\pi}$, $\mathit{evolve}[\![\boldsymbol{\rho}, \boldsymbol{\pi}]\!]$ "switches" its truthfulness and computes all new input environments $\boldsymbol{\rho}'$ that are compatible with the switched constraint. Here, a new input environment $\boldsymbol{\rho}'$ is compatible with $\boldsymbol{\pi}$ if running the user program with $\boldsymbol{\rho}'$ produces a log $\boldsymbol{\pi}'$ that has the same prefix as $\boldsymbol{\pi}$ plus the constraint that *evolve* has switched to obtain $\boldsymbol{\rho}'$. Put differently, *evolve* returns all possible evolutions of the current input that direct the concolic tester to explore a new aspect of the behavior of the user program. Theorem 3 from section 4 states this property formally.

$$\frac{\langle \boldsymbol{\rho}', \boldsymbol{\pi}' \rangle \in \mathit{evolve}[\![\boldsymbol{\rho}, \boldsymbol{\pi}]\!]}{\langle \boldsymbol{\rho}', \boldsymbol{\pi}' \rangle \in \mathit{evolve}[\![\boldsymbol{\rho}, \boldsymbol{\pi} \mathbin{+\!\!+} [\mathbf{p}]]\!]}\;[\text{M-P}\textsc{refix}]$$

$$\frac{\begin{array}{l}\boldsymbol{\pi} = \boldsymbol{\pi}_1 \mathbin{+\!\!+} [\langle \text{“R-C}\textsc{ond}\text{”}, \text{“F}\textsc{alse}\text{”}, \mathbf{v}\rangle] \\ \boldsymbol{\pi}' = \boldsymbol{\pi}_1 \mathbin{+\!\!+} [\langle \text{“R-C}\textsc{ond}\text{”}, \text{“T}\textsc{rue}\text{”}, \mathbf{v}\rangle] \\ \boldsymbol{\rho}' = \mathit{update}[\![\boldsymbol{\rho}, \boldsymbol{\pi}']\!]\end{array}}{\langle \boldsymbol{\rho}', \boldsymbol{\pi}' \rangle \in \mathit{evolve}[\![\boldsymbol{\rho}, \boldsymbol{\pi}]\!]}\;[\text{M-T}\textsc{rue}] \qquad \frac{\begin{array}{l}\boldsymbol{\pi} = \boldsymbol{\pi}_1 \mathbin{+\!\!+} [\langle \text{“R-C}\textsc{ond}\text{”}, \text{“T}\textsc{rue}\text{”}, \mathbf{v}\rangle] \\ \boldsymbol{\pi}' = \boldsymbol{\pi}_1 \mathbin{+\!\!+} [\langle \text{“R-C}\textsc{ond}\text{”}, \text{“F}\textsc{alse}\text{”}, \mathbf{v}\rangle] \\ \boldsymbol{\rho}' = \mathit{update}[\![\boldsymbol{\rho}, \boldsymbol{\pi}']\!]\end{array}}{\langle \boldsymbol{\rho}', \boldsymbol{\pi}' \rangle \in \mathit{evolve}[\![\boldsymbol{\rho}, \boldsymbol{\pi}]\!]}\;[\text{M-F}\textsc{alse}]$$

Figure 13: Negating Conditional Branches in User Programs

Figure 13 collects the three most basic rules of the definition of *evolve*. The first rule, [M-PREFIX], is an administrative one; it allows the removal of an arbitrary suffix from the log $\boldsymbol{\pi}$ so that the rest of the rules can focus on the last entry of the remaining log.

The next two rules, [M-FALSE] and [M-TRUE], form the first-order aspect of *evolve* that we discuss in section 2.1. They fire when the last entry of the log is a path constraint from a branch of a `cond` expression of the user program. Their purpose is to guide *evolve* to generate an input that forces concolic evaluation to change the outcome of the branch. To do so, the two rules replace the constraint with its "negation" and then, with metafunction *update*, they present the modified list of constraints as a problem to an SMT solver and use the solution to obtain a new input environment $\boldsymbol{\rho}'$.

Figure 14 presents the higher-order rules and figure 15 contains the auxiliary definitions they need. The higher-order rules switch an input constraint of form $\langle$“R-CASE”, $\boldsymbol{\ell}$, $\mathbf{v}$, _$\rangle$. Recall that such constraints result from the evaluation of a `case` expression with label $\boldsymbol{\ell}$ in the body of a canonical function. Thus an input

$$\frac{\begin{array}{l}\mathbf{F} \in \mathrm{dom}(\boldsymbol{\rho}),\ \boldsymbol{\rho}(\mathbf{F}) = \mathbf{C}^{\circ}[(\mathtt{case}^{\ell}\ \mathbf{y})] \qquad \boldsymbol{\pi} = \boldsymbol{\pi}_1 +\!\!+ [\langle\text{“R-CASE”}, \ell, (\lambda\mathbf{x}.\ \mathbf{e}), \text{“MISS”}\rangle] \\ \langle \boldsymbol{\rho}_1, \mathbf{e}^{\circ}\rangle \in \mathit{action}_b[\![\boldsymbol{\rho}, \mathit{locals}[\![\mathbf{C}^{\circ}]\!], \{\mathbf{y}\} \cup \mathit{locals}_p[\![\mathbf{C}^{\circ}]\!]]\!] \\ \text{fresh } \ell_1 \notin \mathrm{labels}(\boldsymbol{\rho}_1, \mathbf{e}^{\circ}) \qquad \boldsymbol{\pi}' = \boldsymbol{\pi}_1 +\!\!+ [\langle\text{“R-CASE”}, \ell, (\lambda\mathbf{x}.\ \mathbf{e}), \text{“HIT”}: \ell_1\rangle] \\ \boldsymbol{\rho}' = \boldsymbol{\rho}_1[\mathbf{F} \mapsto \mathbf{C}^{\circ}[(\mathtt{case}^{\ell}\ \mathbf{y}\ [\mathtt{procedure?}\ \mathbf{e}^{\circ}]^{\ell_1})]]\end{array}}{\langle \boldsymbol{\rho}', \boldsymbol{\pi}'\rangle \in \mathit{evolve}[\![\boldsymbol{\rho}, \boldsymbol{\pi}]\!]}\ [\text{M-NEWPROC1}]$$

$$\frac{\begin{array}{l}\mathbf{F} \in \mathrm{dom}(\boldsymbol{\rho}),\ \boldsymbol{\rho}(\mathbf{F}) = \mathbf{C}^{\circ}[(\mathtt{case}^{\ell}\ \mathbf{y})] \qquad \boldsymbol{\pi} = \boldsymbol{\pi}_1 +\!\!+ [\langle\text{“R-CASE”}, \ell, \langle\mathbf{t}\rangle, \text{“MISS”}\rangle] \\ \langle \boldsymbol{\rho}_1, \mathbf{e}^{\circ}\rangle \in \mathit{action}_b[\![\boldsymbol{\rho}, \mathit{locals}[\![\mathbf{C}^{\circ}]\!], \{\mathbf{y}\} \cup \mathit{locals}_p[\![\mathbf{C}^{\circ}]\!]]\!] \\ \text{fresh } \ell_1 \notin \mathrm{labels}(\boldsymbol{\rho}_1, \mathbf{e}^{\circ}) \qquad \boldsymbol{\pi}' = \boldsymbol{\pi}_1 +\!\!+ [\langle\text{“R-CASE”}, \ell, \langle\mathbf{t}\rangle, \text{“MISS”}\rangle] \\ \boldsymbol{\rho}' = \boldsymbol{\rho}_1[\mathbf{F} \mapsto \mathbf{C}^{\circ}[(\mathtt{case}^{\ell}\ \mathbf{y}\ [\mathtt{procedure?}\ \mathbf{e}^{\circ}]^{\ell_1})]]\end{array}}{\langle \boldsymbol{\rho}', \boldsymbol{\pi}'\rangle \in \mathit{evolve}[\![\boldsymbol{\rho}, \boldsymbol{\pi}]\!]}\ [\text{M-NEWPROC2}]$$

$$\frac{\begin{array}{l}\mathbf{F} \in \mathrm{dom}(\boldsymbol{\rho}),\ \boldsymbol{\rho}(\mathbf{F}) = \mathbf{C}^{\circ}[(\mathtt{case}^{\ell}\ \mathbf{y}\ [\mathtt{procedure?}\ \mathbf{e}_1^{\circ}]^{\ell_1}\ [\langle\mathbf{t}_2\rangle\ \mathbf{e}_2^{\circ}]^{\ell_2} \ldots)] \\ \boldsymbol{\pi} = \boldsymbol{\pi}_1 +\!\!+ [\langle\text{“R-CASE”}, \ell, \langle\mathbf{t}\rangle, \_\rangle] \\ \langle \boldsymbol{\rho}_1, \mathbf{e}^{\circ}\rangle \in \mathit{action}_b[\![\boldsymbol{\rho}, \mathit{locals}[\![\mathbf{C}^{\circ}]\!], \mathit{locals}_p[\![\mathbf{C}^{\circ}]\!]]\!] \\ \text{fresh } \ell_{n+1} \notin \mathrm{labels}(\boldsymbol{\rho}_1, \mathbf{e}^{\circ}) \qquad \boldsymbol{\pi}' = \boldsymbol{\pi}_1 +\!\!+ [\langle\text{“R-CASE”}, \ell, \langle\mathbf{t}\rangle, \text{“HIT”}: \ell_{n+1}\rangle] \\ \boldsymbol{\rho}_2 = \boldsymbol{\rho}_1[\mathbf{F} \mapsto \mathbf{C}^{\circ}[(\mathtt{case}^{\ell}\ \mathbf{y}\ [\mathtt{procedure?}\ \mathbf{e}_1^{\circ}]^{\ell_1}\ [\langle\mathbf{t}_2\rangle\ \mathbf{e}_2^{\circ}]^{\ell_2} \ldots [\langle\mathbf{t}\rangle\ \mathbf{e}^{\circ}]^{\ell_{n+1}})]] \\ \boldsymbol{\rho}' = \mathit{update}[\![\boldsymbol{\rho}_2, \boldsymbol{\pi}']\!]\end{array}}{\langle \boldsymbol{\rho}', \boldsymbol{\pi}'\rangle \in \mathit{evolve}[\![\boldsymbol{\rho}, \boldsymbol{\pi}]\!]}\ [\text{M-NEWINT}]$$

$$\frac{\begin{array}{l}\boldsymbol{\pi} = \boldsymbol{\pi}_1 +\!\!+ [\langle\text{“R-CASE”}, \ell, \langle\mathbf{t}\rangle, \_\rangle] \\ \mathbf{F} \in \mathrm{dom}(\boldsymbol{\rho}),\ \boldsymbol{\rho}(\mathbf{F}) = \mathbf{C}^{\circ}[(\mathtt{case}^{\ell}\ \mathbf{y}\ [\mathtt{procedure?}\ \mathbf{e}_1^{\circ}]^{\ell_1}\ [\langle\mathbf{t}_2\rangle\ \mathbf{e}_2^{\circ}]^{\ell_2} \ldots)] \\ [\langle\mathbf{t}_2, \ell_2\rangle, \ldots] = [\langle\mathbf{t}_p, \ell_p\rangle, \ldots] +\!\!+ [\langle\mathbf{t}_i, \ell_i\rangle] +\!\!+ [\langle\mathbf{t}_s, \ell_s\rangle, \ldots] \\ \boldsymbol{\pi}' = \boldsymbol{\pi}_1 +\!\!+ [\langle\text{“R-CASE”}, \ell, \langle\mathbf{t}\rangle, \text{“HIT”}: \ell_i\rangle] \qquad \boldsymbol{\rho}' = \mathit{update}[\![\boldsymbol{\rho}, \boldsymbol{\pi}']\!]\end{array}}{\langle \boldsymbol{\rho}', \boldsymbol{\pi}'\rangle \in \mathit{evolve}[\![\boldsymbol{\rho}, \boldsymbol{\pi}]\!]}\ [\text{M-CHANGE}]$$

Figure 14: Directed Evolution of Higher-order Inputs constraint is sufficient for *evolve* to identify the `case` expression in the input environment it concerns.

Rules [M-NEWPROC1] and [M-NEWPROC2] apply when the `case` expression with label $\ell$ is empty. They modify $\boldsymbol{\rho}$ to extend the `case` expression with a `procedure?` clause, the default first clause for recognizing function arguments. Rule [M-NEWPROC1] handles the situation where $\mathbf{v}$, the value `case` examines, is a function. To create a new clause, [M-NEWPROC1] calls $\mathit{action}_b$ to compute new actions — we return to this metafunction towards the end of the section. Rule [M-NEWPROC2] handles the situation where $\mathbf{v}$ is a first-order concolic value $\langle\mathbf{t}\rangle$. It is the same as [M-NEWPROC1] except that the new list of constraints still ends with $\langle$“R-CASE”, $\ell$, $\mathbf{v}$, “MISS”$\rangle$ as $\langle\mathbf{t}\rangle$ cannot match the new `procedure?` clause of the `case` expression.

If the `case` expression with label $\ell$ is non-empty, the concolic tester can change its evaluation only when $\mathbf{v}$ is not a function. After all, if $\mathbf{v}$ is a function, the evaluation of a non-empty `case` always follows the first clause of the `case`. As

$\mathbf{C}^\circ$ is the compatible context of $\mathbf{e}^\circ$.

$\Delta \subset \{\mathbf{x}, \mathbf{y}, \mathbf{z}, \dots\}$ stands for any finite subset of non-concolic variables.

$\mathit{locals} : \mathbf{C}^\circ \longrightarrow \Delta$
Given a compatible context of canonical functions, computes the set of all variables in scope in the hole.

$\mathit{locals}_p : \mathbf{C}^\circ \longrightarrow \Delta$
Given a compatible context of canonical functions, compute the set of all variables in scope in the hole that are bound to functions.

$$\frac{\langle \rho', \mathbf{v}^\circ \rangle \in \mathit{action}_c[\![\rho, \Delta]\!] \quad \mathbf{f} \in \Delta_p \quad \text{fresh } \mathbf{x} \notin \Delta \quad \text{fresh } \ell \notin \text{labels}(\rho, \mathbf{v}^\circ)}{\langle \rho', \texttt{let } \mathbf{x} = \mathbf{f}\ \mathbf{v}^\circ \texttt{ in } (\texttt{case}^\ell\ \mathbf{x}) \rangle \in \mathit{action}_b[\![\rho, \Delta, \Delta_p]\!]} \text{[E-Havoc]}$$

$\boxed{\mathit{action}_b : \rho\ \Delta\ \Delta \longrightarrow [\langle \rho, \mathbf{e}^\circ \rangle, \ldots]}$

$$\frac{\langle \rho', \mathbf{v}^\circ \rangle \in \mathit{action}_c[\![\rho, \Delta]\!]}{\langle \rho', \mathbf{v}^\circ \rangle \in \mathit{action}_b[\![\rho, \Delta, \Delta_p]\!]} \text{[E-Const]}$$

$\boxed{\mathit{action}_c : \rho\ \Delta \longrightarrow [\langle \rho, \mathbf{v}^\circ \rangle, \ldots]}$

$$\frac{\text{fresh } \mathbf{X} \notin \text{dom}(\rho)}{\langle \rho[\mathbf{X} \mapsto 0], \mathbf{X} \rangle \in \mathit{action}_c[\![\rho, \Delta]\!]} \text{[C-Int1]} \qquad \frac{\mathbf{x} \in \Delta}{\langle \rho, \mathbf{x} \rangle \in \mathit{action}_c[\![\rho, \Delta]\!]} \text{[C-Bound]}$$

$$\frac{\mathbf{X} \in \text{dom}(\rho),\ \rho(\mathbf{X}) = \mathbf{n}}{\langle \rho, \mathbf{X} \rangle \in \mathit{action}_c[\![\rho, \Delta]\!]} \text{[C-Int2]} \qquad \frac{\text{fresh } \mathbf{x} \notin \Delta \quad \text{fresh } \ell \notin \text{labels}(\rho)}{\langle \rho, \lambda \mathbf{x}.\ (\texttt{case}^\ell\ \mathbf{x}) \rangle \in \mathit{action}_c[\![\rho, \Delta]\!]} \text{[C-Proc]}$$

Figure 15: Computation of New Actions & Local Variables we discuss in section 2.3 and section 2.4, if **v** is a first-order concolic value ‹**t**›, the tester has two options: either to extend the case expression with a new clause, or to assert that ‹**t**› matches an existing clause. Rules [M-NewInt] and [M-Change] handle these two cases, respectively. There are two subcases for [M-NewInt]: ‹**t**› matches an existing clause but the tester opts to create a dedicated clause for it in the next iteration of the concolic loop, or ‹**t**› does not match any existing clause and the tester extends the case to accommodate it. In either case, rule [M-NewInt] computes the new actions for the additional clause in the same manner as in [M-NewProc1] and the new clause is inserted into the case expression. As a last step, rule [M-NewInt] queries the SMT solver to adjust the values of first-order inputs in the environment, ensuring that all the clauses of the extended case are distinct. Rule [M-Change] corresponds to the discussion in section 2.4 and its goal is to assert that ‹**t**› matches an existing clause $\ell_i$ of the case expression. Hence *evolve* replaces the last entry of the log with ⟨"R-Case", $\ell$, ‹**t**›, "Hit": $\ell_i$⟩. Similar to the previous rule, as a last step rule [M-Change] consults the SMT solver to adjust the input environment given the new constraint about ‹**t**›.

As a final remark, metafunction $\mathit{action}_b$ computes the set of actions for the new case clauses that *evolve* introduces. It largely follows the grammar of $\mathbf{e}^\circ$ discussed in section 3.1. When it introduces a new function or a let-expression as a new action, $\mathit{action}_b$ constructs an empty case for their corresponding body expressions. Moreover, $\mathit{action}_b$ delegates to *locals* and $\mathit{locals}_p$ to compute the set of variables that new actions can refer to. The metafunction *locals* takes a context $\mathbf{C}^\circ$ and extracts the set of all local variables visible in the hole. The metafunction $locals_p$ is similar to *locals* but only extracts variables that are bound to functions.

#### 3.3 Adding Concretization

$$\underline{\mathbf{e}} ::= \ldots . \mid \mathtt{concretize}(\underline{\mathbf{e}}) \qquad \mathbf{e} ::= \ldots . \mid \mathtt{concretize}(\mathbf{e})$$

$$\frac{\mathbf{n} = \mathcal{E}[\![\boldsymbol{\rho}, \mathbf{t}]\!]}{\langle \boldsymbol{\rho}, \boldsymbol{\pi}, \mathtt{concretize}(\langle \mathbf{t} \rangle)\rangle \longrightarrow \langle \boldsymbol{\rho}, \boldsymbol{\pi}, \langle \mathbf{n} \rangle \rangle} \text{[R-CONCRETIZE]}$$

Figure 16: Adding Concretization to Concolic Evaluation

Figure 16 shows the extensions for concretization. For simplicity, we identify concrete values with ‹**n**› and consider such terms as feasible to interoperate with external functions. We do not introduce any specific concrete evaluation rules. Instead, we augment the reduction rules of the concolic machine with the [R-CONCRETIZE] that reduces the new form, `concretize(‹t›)`, to its concrete counterpart with the help of $\mathcal{E}$. Recall that the latter metafunction uses the current input $\boldsymbol{\rho}$ to compute the value of the formula $\mathbf{t}$ of a concolic value.

```
date<     = λd1. λd2. (or ((date-year d1) < (date-year d2)) ···)
main-bad  = λdates. (let sorted-dates = (sort dates date<) in ···)
sort/wrap = λlst. λcmp. (sort lst (λx. λy. concretize(cmp x y)))
main-ok   = λdates. (let sorted-dates = (sort/wrap dates date<) in ···)
```

Figure 17: sort With Concretization Wrapper

The astute reader will have noticed that the concretization extension handles only first-order values. In the remainder of the section, by revisiting the example from section 1 in figure 17, we argue informally that in fact this is sufficient, even for functions. In the example, `date<` is a buggy comparison function and `sort` is a library function that is polymorphic in its list argument. Since `sort` is external to the concolic tester, the evaluation of its body is delegated to a concrete machine which does not record constraints nor handles concolic values. This quickly becomes an issue for testing `main-bad`. To discover the bug, the concolic machine needs to log constraints from the evaluation of `date<` and `main-bad`. However, this implies that `date<` produces concolic values which flow to `sort` and disrupt the concrete evaluation of its body.

A straightforward non-solution is to fully concretize the list of dates and miss recording the critical path constraints from the evaluation of `date<`'s body. In contrast, our approach enables both the seamless interoperation of the concolic tester with external libraries and the collection of constraints. The key insight is to create wrappers that strategically `concretize` concolic values. By assumption, `sort` is parametric to its input list. Thus `sort` can consume a list of concolic values as long as the comparison function produces concrete results. This leads to the `sort/wrap` function that behaves like `sort`, except that its `cmp` argument is wrapped in a function that concretizes `cmp`'s return value.

The mechanism for creating correct wrappers for higher-order constructs from user annotations is well-studied [13, 16, 40], thus we do not formalize it. However, we note that our proof-of-concept implementation, discussed in section 5, supports all the necessary features to run the example of this section including lists, external functions, concretization annotations and interoperability between a concrete and a concolic machine.

### 4 Correctness of Higher-order Concolic Testing

This section establishes three facts about our concolic tester that together entail its correctness. First, given an input, if concolic evaluation of a user program triggers an `error` so does the concrete evaluation of the program (soundness). Second, relative to the completeness of SMT solvers, the concolic tester always manages to produce an input in canonical form that triggers `error` in the user program, if a counter-example for the program exists (completeness). Third, for each iteration of the concolic loop, the concolic tester produces a new input that explores a specific and selected-in-advance aspect of the behavior of the user program (directness). Here we discuss the necessary bits for the formal statements of the three facts. The complete formal development with all the proofs are at `https://github.com/shhyou/chop-esop-supplementary`.

Soundness guarantees that the concolic machine respects the semantics of user programs. Thus, the information that the concolic machine logs or its use of concolic values do not affect the evaluation of programs. Specifically, the soundness theorem states that if the concolic evaluation of user program $\underline{\mathbf{e}}$ with proper input environment $\boldsymbol{\rho}$ reduces to `error`,[2] the concrete evaluation of $\underline{\mathbf{e}}$ with $\boldsymbol{\rho}$ also reduces to `error`. Since `error` represents bugs in the user program, soundness effectively reassures that concolic evaluation does not discover spurious bugs.

For the formal statement of the theorem, we first introduce a few technical devices. For closed user programs, i.e., those without input or other free variables, we define a standard call-by-value reduction semantics with reduction relation $\longrightarrow_{\lambda}$. Let $\mathcal{C}[\![\boldsymbol{\rho}, \underline{\mathbf{e}}]\!]$ be the metafunction that constructs concrete inputs from the input environment $\boldsymbol{\rho}$ and substitutes them in $\underline{\mathbf{e}}$. That is, $\mathcal{C}$ traverses the user program $\underline{\mathbf{e}}$, dropping any `concretize` forms and, for each $\mathbf{X}$ in $\underline{\mathbf{e}}$, if $\boldsymbol{\rho}$ maps $\mathbf{X}$ to a number, $\mathcal{C}$ replaces $\mathbf{X}$ with the number. Otherwise if $\boldsymbol{\rho}$ maps $\mathbf{X}$ to a function, $\mathcal{C}$ compiles the canonical function into an equivalent concrete function and replaces $\mathbf{X}$ with the result.

[2] An environment $\boldsymbol{\rho}$ is *proper* if (i) it maps all concolic variables occurring free in canonical functions in $\boldsymbol{\rho}$ to numbers, (ii) all labels in $\boldsymbol{\rho}$ are unique and (iii) the concrete counterparts of the tests of the clauses in `case` expressions are numbers. In this section, we only consider proper environments.

**Theorem 1 (Soundness).** *Let* $\underline{\mathbf{e}}$ *be any user program written in the extended language from section 3.3, i.e.* $\underline{\mathbf{e}}$ *with* concretize *forms. Let* $\boldsymbol{\rho}$ *be any input environment closing* $\underline{\mathbf{e}}$*. If* $\langle \boldsymbol{\rho}, [], \mathcal{L}[\![\boldsymbol{\rho}, \underline{\mathbf{e}}]\!]\rangle \longrightarrow^* \langle \boldsymbol{\rho}, \boldsymbol{\pi}, \mathsf{error}\rangle$ *then* $\mathcal{C}[\![\boldsymbol{\rho}, \underline{\mathbf{e}}]\!] \longrightarrow_{\lambda}^* \mathsf{error}$.

Completeness captures that if the concrete evaluation of a user program with some input raises error, our concolic tester can find the input through the iterative evolution of initially default inputs. More precisely, Theorem 2 formalizes the iterative evolution process as a sequence of pairs of inputs and logs $\langle \boldsymbol{\rho}_1, \boldsymbol{\pi}_1\rangle, \ldots, \langle \boldsymbol{\rho}_m, \boldsymbol{\pi}_m\rangle$ such that (i) the sequence starts with an input environment that contains numbers and default canonical functions and ends with an input environment that triggers error; (ii) each $\boldsymbol{\pi}_i$ is the log produced by the concolic evaluation of the user program with input environment $\boldsymbol{\rho}_i$, and (iii) most importantly, each and every adjacent pairs in the sequence is connected by *evolve*: $\langle \boldsymbol{\rho}_{i+1}, \boldsymbol{\pi}'\rangle \in \mathit{evolve}[\![\boldsymbol{\rho}_i, \boldsymbol{\pi}_i]\!]$ and $\boldsymbol{\pi}'$ is equivalent to a prefix of $\boldsymbol{\pi}_{i+1}$. In particular, conclusion (iii) says that using the logs from each iteration, *evolve* predicts the logs for the next iteration.

**Theorem 2 (Completeness).** *For any* $\underline{\mathbf{e}}$ *written in the user language in section 3.1 with concolic variables* $\mathbf{X}_1, \ldots, \mathbf{X}_n$*, if there exists closed values* $\underline{\mathbf{v}}_1, \ldots, \underline{\mathbf{v}}_n$ *in the language of user programs such that none of the values contain* error *and* $\underline{\mathbf{e}}\{\mathbf{X}_1 \mapsto \underline{\mathbf{v}}_1, \ldots\} \longrightarrow_{\lambda}^* \mathsf{error}$ *then there exists a sequence of environments and logs* $\langle \boldsymbol{\rho}_1, \boldsymbol{\pi}_1\rangle, \ldots, \langle \boldsymbol{\rho}_m, \boldsymbol{\pi}_m\rangle$ *such that* $\mathit{dom}(\boldsymbol{\rho}_1) = \{\mathbf{X}_1, \ldots, \mathbf{X}_n\}$ *and*

1. *For all* $\mathbf{X} \in \mathit{dom}(\boldsymbol{\rho}_1)$*, either* $\boldsymbol{\rho}_1(\mathbf{X}) = 0$ *or* $\boldsymbol{\rho}_1(\mathbf{X}) = \lambda\mathbf{x}.\, (\mathsf{case}^{\ell}\ \mathbf{x})$.
2. *For all* $1 \leqslant i < m$, $\langle \boldsymbol{\rho}_i, [], \mathcal{L}[\![\boldsymbol{\rho}_i, \underline{\mathbf{e}}]\!]\rangle \longrightarrow^* \langle \boldsymbol{\rho}_i, \boldsymbol{\pi}_i, \mathbf{e}_i\rangle$.
3. *For all* $1 \leqslant i < m$*, there exists a pair* $\langle \boldsymbol{\rho}_{i+1}, \boldsymbol{\pi}'_{i+1}\rangle \in \mathit{evolve}[\![\boldsymbol{\rho}_i, \boldsymbol{\pi}_i]\!]$ *such that* $\boldsymbol{\pi}'_{i+1}$ *is equivalent to a prefix of* $\boldsymbol{\pi}_{i+1}$.
4. $\langle \boldsymbol{\rho}_m, [], \mathcal{L}[\![\boldsymbol{\rho}_m, \underline{\mathbf{e}}]\!]\rangle \longrightarrow^* \langle \boldsymbol{\rho}_m, \boldsymbol{\pi}_m, \mathsf{error}\rangle$.

There are two points worth unpacking here. First, conclusion 1 assumes an appropriate choice between numbers and default canonical functions in the initial environment $\boldsymbol{\rho}_1$. In an implementation, either the user supplies an input specification or the tester employs some sophisticated search strategy over all combinations. Second, since the user program may diverge, in conclusion 2 the concolic machine may need to end the evaluation early. As the maximum number of steps needed is finite, an implementation can overcome this by setting a time limit.

We prove Theorem 2 in two steps. First, we show that if there is an input for which the concrete evaluation of a user program raises error, then there exists an input environment $\boldsymbol{\rho}$ that contains numbers and canonical functions that also causes the concolic machine to triggers an error. Thus this step validates the definition of canonical functions.

**Lemma 1 (Representation Completeness).** *We say that* $\langle \boldsymbol{\rho}, \boldsymbol{\pi}\rangle$ *is a* proper counterexample *for a user program* $\underline{\mathbf{e}}$ *if (i)* $\boldsymbol{\rho}$ *closes* $\underline{\mathbf{e}}$*, i.e.* $FV(\underline{\mathbf{e}}) \subset \mathit{dom}(\boldsymbol{\rho})$*, (ii)* $\langle \boldsymbol{\rho}, [], \mathcal{L}[\![\boldsymbol{\rho}, \underline{\mathbf{e}}]\!]\rangle \longrightarrow^* \langle \boldsymbol{\rho}, \boldsymbol{\pi}, \mathsf{error}\rangle$ *and (iii)* $\boldsymbol{\pi}$ *does not contain input constraints of the form* ⟨"R-Case", $\boldsymbol{\ell}$, $\mathbf{v}$, "Miss"⟩.

*For any user program* $\underline{\mathbf{e}}$ *with inputs* $\mathbf{X}_1, \ldots, \mathbf{X}_n$. *if there exists closed values* $\underline{\mathbf{v}}_1, \ldots, \underline{\mathbf{v}}_n$ *such that no value contains* `error` *and* $\underline{\mathbf{e}}\{\mathbf{X}_1 \mapsto \underline{\mathbf{v}}_1, \ldots\} \longrightarrow_\lambda^* \texttt{error}$ *then there exists a proper counterexample of* $\underline{\mathbf{e}}$.

In the second step of the proof of Theorem 2, we show that the evolution of inputs during the concolic loop results in an environment input that can trigger an `error` if such an input exists. As a consequence, the concolic tester only needs to explore inputs it generates with *evolve*.

**Lemma 2 (Search Completeness).** *For any* $\underline{\mathbf{e}}$ *with inputs* $\mathbf{X}_1, \ldots, \mathbf{X}_n$, *if* $\underline{\mathbf{e}}$ *has a proper counterexample then there exists a sequence of environments and logs satisfying Theorem 2 (1)–(4).*

The last fact we establish for our concolic tester is necessary for the proof of Lemma 2, but also has value on its own. It entails that, at each iteration of the concolic loop, the concolic tester aims to explore a specific aspect of the behavior of the user program and indeed produces new inputs that achieve this goal. We call this the *concolic property*. Formally, Theorem 3 shows that after the concolic machine evaluates a user program with an input environment produced by *evolve*, the machine's log is a prefix of the log *evolve* predicts.

**Theorem 3 (Concolic).** *For any* $\underline{\mathbf{e}}$ *and* $\boldsymbol{\rho}_1$, *if*

1. $\langle \boldsymbol{\rho}_1, [\,], \mathcal{L}[\![\boldsymbol{\rho}_1, \underline{\mathbf{e}}]\!]\rangle \longrightarrow^* \langle \boldsymbol{\rho}_1, \boldsymbol{\pi}_1 +\!\!+ [\mathbf{p}_1], \mathbf{e}_1\rangle$.
2. $\boldsymbol{\pi}_1$ *has no "miss" input constraints (of the form* $\langle$"R-CASE", $\boldsymbol{\ell}$, $\mathbf{v}$, "MISS"$\rangle$*).*
3. $\langle \boldsymbol{\rho}_2, \boldsymbol{\pi}_1 +\!\!+ [\mathbf{p}]\rangle \in evolve[\![\boldsymbol{\rho}_1, \boldsymbol{\pi}_1 +\!\!+ [\mathbf{p}_1]]\!]$.

*then* $\langle \boldsymbol{\rho}_2, [\,], \mathcal{L}[\![\boldsymbol{\rho}_2, \underline{\mathbf{e}}]\!]\rangle \longrightarrow^* \langle \boldsymbol{\rho}_2, \boldsymbol{\pi}_2 +\!\!+ [\mathbf{p}_2], \mathbf{e}_2\rangle$ *such that* $\boldsymbol{\pi}_1 +\!\!+ [\mathbf{p}]$ *is equivalent to* $\boldsymbol{\pi}_2 +\!\!+ [\mathbf{p}_2]$.

### 5 From the Model to a Proof-of-Concept Implementation

A question about our model is whether it can serve as a guide for an effective higher-order concolic tester. To provide some positive evidence, we have implemented a prototype that closely follows the model. The prototype plays the role of a sanity check that our theoretically-correct model is not inherently impractical; performance was not a serious concern. Notably, the prototype's input generation strategy is naive. To ensure progress, the prototype sets a configurable timeout for each run and avoids duplicating work with a log from trying each input it generates. We leave the details to `https://github.com/shhyou/chop-esop-supplementary` and only summarize our experimental results here.

We compiled a benchmark suite from three sources. The primary source is Nguyễn et al. [33]'s work, specifically from the `jfp` branch of `https://github.com/philnguyen/soft-contract`. These programs ultimately come from other papers; see figure 18. The second source is CutEr [18], the Erlang concolic tester. We collected all of the test cases in CutEr's test suite that use higher-order functions and translated them to our prototype's language. Finally, we contribute three small examples as as part of this work that have proven out of reach for

| Name | Failures | Source |
|---|---|---|
| games | 3/3 | Nguyễn et al. [32, 33] |
| hors | 0/23 | Kobayashi et al. [27] |
| mochi-new | 0/11 | Kobayashi et al. [27] |
| octy | 0/13 | Tobin-Hochstadt and Felleisen [44] |
| others | 1/26 | Nguyễn et al. [32, 33], Tobin-Hochstadt and Van Horn [45] |
| softy | 0/12 | Cartwright and Fagan [10], Wright and Cartwright [46] |
| terauchi | 0/7 | Terauchi [42] |
| cuter | 0/20 | Giantsios et al. [18] |
| c-hop | 0/3 | Interesting examples we discovered |
| **total** | **4/118** | |

Figure 18: Benchmark Results both Nguyễn et al. [33]'s tool and CutEr. Overall, the benchmark programs use the Scheme numeric tower, booleans, lists, objects encoded as functions [1], strings, symbols, and higher-order functions.

Out of 118 benchmarks, our prototype fails to discover bugs in 4 of the programs. These programs can be grouped based on two limitations of our prototype. First, our search strategy is naive and as a result two benchmarks time out after an hour. Second, our prototype does not handle Racket's `struct` declaration and a few other complex syntactic features of Racket that two of Nguyễn et al. [33]'s benchmarks use.

### 6 Related Work

**Concolic Testing.** CutEr [17, 18] is a concolic testing tool for Erlang [4]. Although CutEr generates functions, it does not generate inputs that contain calls in their bodies.[3] Palacios and Vidal [34] offer an instrumentation approach for concolic testers of functional languages but do not address the generation of higher-order inputs.

Li et al. [31] extend the design of path constraints with symbolic subtype expressions to handle polymorphism in object-oriented languages. However, their input generation uses only already defined classes.

Path explosion remains a central challenge for concolic testing techniques [5, 9], and it is a challenge that has lead to approaches that rely on the correct handling of function inputs. Godefroid [19] compute function summaries on-the-fly to tame the combinatorial explosion of the search space of control-flow paths. Similarly, Anand et al. [2] performs symbolic execution compositionally using function summaries. FOCAL [24] breaks programs down into small units to reduce the search space; it tests each units individual and constructs a system-level tests by using summaries. In all three cases, the summaries are first-order and do not include higher-order interactions between functions.

[3] Personal communication with Kostis Sagonas.

**Higher-order Symbolic Execution.** Nguyễn et al. [33] and Tobin-Hochstadt and Van Horn [45] propose the idea of refining symbolic unknown values into canonical shapes to generate higher-order counterexamples. We adapt their refinement rules into the grammar of canonical functions in figure 10. Unfortunately, despite opposite claims, their rules are not complete and fail to generate a counter-example for our buggy `call-twice` from section 1.[4] Our work provably fixes this issue. Moreover, we introduce the notion of input constraints to support the directed search of the higher-order input space.

**Random Testing.** QuickCheck [11] supports random testing of higher-order functions by using user-provided maps from the input type to integers and from integers to the output type. Koopman and Plasmeijer [28] improves upon QuickCheck by using a predefined datatype to represent the syntax of higher-order functions. LambdaTester [36] focuses on testing and generating higher-order functions that mutate an object's state in order to affect control-flow paths that depend on this state. Klein et al. [26] random-generate higher-order inputs that call their arguments to trigger bugs in stateful programs with opaque types.

### 7 Conclusion

This work offers a theoretical roadmap for generalizing concolic testing to programs with higher-order inputs. The central innovation is that our concolic tester records salient information about the interactions between a user program and its (canonical) inputs. The information induces an SMT problem that describes a new canonical input that exercises a yet unexplored aspect of the user program.

For this paper, we focus on the quintessential higher-order linguistic feature, higher-order functions. That said, much remains to be done to build this theory into a production tool by, for example, using the insights of this paper to support other features such as objects and mutable state. Specifically for state, our model can be easily and soundly extended to imperative user programs. However, completeness and the generation of stateful function inputs requires further study. Finally, another important direction is improving the implementation, notably exploring search optimizations and strategies. Our prototype uses a naive strategy and this hampers its performance. Nevertheless, we view this paper an essential first step towards sophisticated testing strategies for modern programming languages.

**Acknowledgments** We appreciate Phúc C. Nguyễn, Sam Tobin-Hochstadt, David Van Horn, Aggelos Giantsios, Nikolaos Papaspyrou and Konstantinos Sagonas for explaining the details of their work and being an inspiration for ours. We thank Spencer P. Florence, Lukas Lazarek, Wung Jae Lee, Alex Owens, Peter Zhong, and the anonymous reviewers of their thoughtful feedback on earlier versions of this paper. This material is based upon work supported by the National Science Foundation under Grant No. CNS-1823244.

[4] Personal communication with Phúc Nguyễn.
